# Ingestion of Isobutyl Nitrite Leading to Methemoglobinemia: A Case Report

**DOI:** 10.7759/cureus.97471

**Published:** 2025-11-21

**Authors:** Drake D Dixon, Mohammed Rahman, Hanna Nour, Larissa Dub

**Affiliations:** 1 Emergency Medicine, University of Central Florida/HCA Florida Osceola Hospital, Kissimmee, USA; 2 Emergency Medicine, University of Central Florida School of Medicine, Orlando, USA; 3 Emergency Medicine, HCA Florida Osceola Hospital, Kissimmee, USA

**Keywords:** amyl nitrite, cyano-methemoglobin pathway, methemoglobinemia, methylene blue, nitrite poisoning

## Abstract

This case report highlights an occurrence of methemoglobinemia caused by the ingestion of liquid amyl nitrite, a substance often misused as a recreational drug. The patient in this case presented with cyanosis and elevated methemoglobin levels. Prompt administration of methylene blue successfully reversed the methemoglobinemia by reducing methemoglobin back to hemoglobin. This case emphasizes the importance of rapid diagnosis and treatment in nitrite-induced methemoglobinemia, demonstrating methylene blue as a highly effective antidote. It also highlights the risks associated with amyl nitrite misuse and the crucial role of healthcare providers in managing this potentially life-threatening condition.

## Introduction

Methemoglobinemia is a rare but potentially life-threatening condition characterized by an increased level of methemoglobin in the blood, a form of hemoglobin that is unable to bind and transport oxygen effectively [1]. Normally, methemoglobin levels are maintained at less than 1% of total hemoglobin through enzymatic reduction pathways [2]. However, exposure to certain chemicals or drugs, including nitrates and nitrites, can overwhelm these pathways, leading to a significant rise in methemoglobin levels and resulting in tissue hypoxia despite normal oxygen saturation readings [3]. If high levels of methemoglobinemia are left unchecked, patients may suffer from severe central nervous system and cardiac dysfunction, and, ultimately, death [2].

One of the most well-documented causes of acquired methemoglobinemia is the ingestion or inhalation of nitrites, such as amyl nitrite or isobutyl nitrite [4]. Isobutyl nitrite and amyl nitrite are commonly used as recreational drugs, known colloquially as “poppers,” but accidental or intentional ingestion can result in severe toxicity [5]. These substances cause severe toxicity by acting as a powerful oxidizing agent in the blood. In healthy red blood cells, the iron atom in the center of the hemoglobin molecule must be in the ferrous state (Fe^2+^) to bind and transport oxygen effectively. When isobutyl nitrite or amyl nitrite enters the bloodstream, it oxidizes the iron, converting the iron from the ferrous state (Fe^2+^) to the ferric state (Fe^3+^) [6]. This oxidized form of hemoglobin is called methemoglobin, which cannot transport oxygen to the tissues effectively. The mainstay of treatment for methemoglobinemia is the administration of methylene blue, which acts as an electron donor to accelerate the reduction of methemoglobin back to hemoglobin, effectively reversing the hypoxic effects [6].

This case report details the presentation and treatment of a patient who ingested liquid amyl nitrite, leading to methemoglobinemia, and was successfully treated with methylene blue.

## Case presentation

A 55-year-old male with a past medical history of early Alzheimer’s, hypertension, and alcohol abuse disorder presented after ingesting 30 mL of isobutyl nitrite. The patient was found by his family unresponsive, with vomit and diarrhea surrounding him. Emergency Medical Services arrived on scene and found the patient to be hypotensive to 70s systolic and hypoxic with SpO_2_ in the 70s. En route, the patient’s hypotension resolved with fluids, but the hypoxia did not correct with supplemental oxygen. The patient arrived at the emergency department alert and oriented, with mild cyanosis of the lips and fingers, and SpO_2_ was 87% while receiving 15 L of supplemental oxygen. The patient stated he drank the isobutyl nitrite, thinking it was alcohol, in an effort to become intoxicated. The patient denied headaches, vision changes, focal weakness, throat pain, chest pain, shortness of breath, fevers, chills, cough, flank pain, or dysuria.

The physical examination was significant for a male patient who was awake, alert, and in no acute distress. The heart rate was initially tachycardic to 110 beats/minute, and the lips and fingers were cyanotic. However, by the time the EKG was obtained, the heart rate had returned to normal limits. There were no motor or sensory deficits, and the patient was not suicidal or homicidal. The rest of the physical examination was unremarkable.

**Figure 1:**
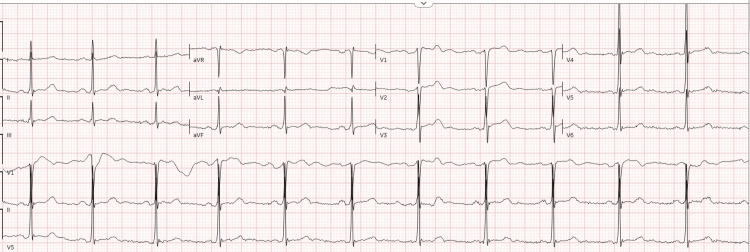
EKG of the patient. EKG obtained with a paper speed of 25 mm/s and a gain of 10 mm/mV.

The workup included a complete blood count (CBC), comprehensive metabolic panel (CMP), alcohol/acetaminophen/salicylate levels, lactate level, blood cultures, chest X-ray, EKG, and urine drug screen (UDS). The CBC, CMP, alcohol/acetaminophen/salicylate levels, lactate level, and EKG can be seen in Table 1 and Figure 1. The EKG showed a normal sinus rhythm with a ventricular rate of 68 beats/minute; the intervals were within normal limits. There were no significant ST-segment or T-wave abnormalities, pathologic Q waves, or conduction delays. Poison control was contacted, who recommended obtaining an arterial blood gas (ABG) with co-oximetry for concern of methemoglobinemia.

**Table 1:** CBC, CMP, lactate levels, and alcohol/acetaminophen/salicylate levels of the patient. Bolded items represent abnormal values. WBC = white blood cells; BUN = blood urea nitrogen; AST = aspartate aminotransferase; ALT = alanine aminotransferase

Laboratory item	Result	Reference range
WBC	11.9	4.0–10.5 × 10^3^/µL
Hemoglobin	13.8	3.7–17.5 g/dL
Hematocrit	39.9	40.1–51.0%
Platelet count	225	150–400 × 10^3^/µL
Sodium	139	136–145 mmol/L
Potassium	3.8	3.7–5.1 mmol/L
Chloride	107	98–107 mmol/L
Carbon dioxide	23.6	21–32 mmol/L
BUN	9	7–18 mg/dL
Creatinine	0.95	0.55–1.3 mg/dL
Glucose	105	74–106 mg/dL
Calcium	9	8.4–10.1 mg/dL
Total bilirubin	0.6	0.3–1.2 mg/dL
AST	22	10–37 U/L
ALT	12	12–78 U/L
Total protein	6.9	6.4–8.2 g/dL
Albumin	4.3	3.4–5.0 g/dL
Lactic acid	2.48	0.4–2.0 mmol/L
Alcohol level	<3	<5 mg/dL
Salicylate level	<3	4–20 mg/dL
Acetaminophen level	<2	10–30 µg/mL

Initial co-oximetry results (Table 2) were significant for a hemoglobin level of 13.2 g/dL, oxyhemoglobin level of 23%, carboxyhemoglobin level of 0%, and methemoglobin level of 44%. Poison control was again contacted with these results, who recommended 2 mg/kg methylene blue due to methemoglobin above 20%. They also recommended a repeat dose in one hour if no improvement in the methemoglobin level was noted, and intensive care unit (ICU) admission for close monitoring.

**Table 2:** Serial co-oximetry levels of the patient. Day 0 refers to the day the patient was evaluated in emergency department and, day 1 refers to the first day of admission in the hospital. The day is followed by the time in 24-hour time format. Hb = hemoglobin; HbO_2_ = oxyhemoglobin; CoHb = carboxyhemoglobin; MetHb = methemoglobin

Laboratory Item	Day 0, 19:36	Day 0, 22:00	Day 1, 00:23	Reference range
Hb level	13.2	12.2	12.9	14.0–18.0 g/dL
HbO_2 _saturation	23	92.8	96.2	92–100%
CoHb level	0	0.4	2.4	0.0–0.8%
MetHb level	44	6.9	0.8	0.2–0.6%

Other laboratory findings were significant for a mild leukocytosis to 11.9 x 10^3^ cells/µL and lactic acid 2.33 mmol/L, but were otherwise unremarkable (Table 1). Chest X-ray showed clear lungs. The ICU was contacted, which accepted the patient for admission. While in the ICU, the patient’s methemoglobin percentage dropped from 44% to 6.9%, then to 0.8% (Table 2). The patient was subsequently downgraded and eventually discharged from the hospital on hospital day two with no significant neurologic or cardiac sequelae.

## Discussion

Previous studies have attempted to evaluate the incidence of acquired methemoglobinemia; however, the exact incidence remains unknown. Some studies rely on the administration of methylene blue to identify cases; however, this may exclude cases that are not treated with methylene blue [4,7]. Therefore, continued documentation of methemoglobinemia cases and their treatment is essential to expand the knowledge base for this condition.

Methemoglobinemia is a life-threatening condition that causes hypoxia, which can progress to organ failure and eventually death. It can be induced by numerous substances, including, but not limited to, antibiotics, nitrates, and anesthetics. Some notable substances include lidocaine, benzocaine, bupivacaine, nitric oxide, nitroglycerin, dapsone, rifampin, and primaquine, among others [6]. Methemoglobinemia should always be considered in the differential diagnosis for patients presenting with unexplained cyanosis or hypoxia unresponsive to supplemental oxygen.

The severity of symptoms depends on the level of methemoglobin, reported as a percentage of total hemoglobin [6]. Levels below 10% are typically asymptomatic, while levels exceeding 70% may be fatal [2]. Symptoms most commonly emerge at intermediate levels. Patients often present with signs and symptoms of hypoxia, including cyanosis, weakness, dyspnea, headache, lightheadedness, dizziness, confusion, and, in severe cases, seizures or coma. Notably, pulse oximetry readings may appear normal or only mildly decreased, which can complicate diagnosis.

Once methemoglobinemia is suspected, an ABG with co-oximetry should be ordered to assess for the presence of methemoglobin, which can sometimes cause a pathognomonic chocolate brown color in the blood [8]. As methemoglobin levels rise, pulse oximeter readings may stabilize and fail to accurately reflect the true degree of hypoxia. Therefore, an ABG is essential to confirm the diagnosis and determine the exact methemoglobin level in the blood.

Once the diagnosis of methemoglobinemia is confirmed, treatment and management involve discontinuing exposure to the precipitating substance, contacting regional poison control, administering supplemental oxygen, and administering methylene blue based on methemoglobin levels and specific contraindications [8]. Typically, treatment with methylene blue is initiated at methemoglobin levels greater than 20% as this is the level where patients are usually symptomatic [1,6]. Methylene blue is contraindicated in cases of glucose-6-phosphate dehydrogenase (G6PD) deficiency. Methylene blue can induce hemolysis in patients with G6PD deficiency. Therefore, treatment with ascorbic acid is recommended [9]. If a patient is taking serotonergic medications, methylene blue should be avoided as it is a potent reversible monoamine oxidase inhibitor. Methylene blue prevents the normal metabolism of serotonin, allowing serotonin levels to rise to potentially dangerous magnitudes [10].

Interestingly, the patient had a small but insignificant increase in carboxyhemoglobin levels after treatment with methylene blue (Table 2). This increase is likely a metabolic consequence of the original toxin, not a direct side effect of the methylene blue [6].

## Conclusions

Methemoglobinemia is a critical, potentially fatal condition demanding immediate clinical recognition and treatment. Clinicians must maintain a high index of suspicion for this diagnosis in any patient presenting with cyanosis or hypoxia that is unresponsive to supplemental oxygen, as standard pulse oximetry is unreliable in this setting. This case underscores the need for continuous efforts in research, education, and reporting to enhance the understanding and management of methemoglobinemia. Expanding the body of evidence through ongoing documentation will help elucidate its true incidence, refine risk stratification, and determine the optimal protocols for both standard and alternative therapies. Furthermore, increased public and professional awareness regarding common precipitating agents is crucial for preventative strategies. Collaborative initiatives between clinicians and toxicologists are vital to bridging current knowledge gaps and developing more effective, evidence-based guidelines, ultimately leading to improved patient outcomes for this life-threatening condition.
